# Health-Related Quality of Life of the Roma in Greece: The Role of Socio-Economic Characteristics and Housing Conditions

**DOI:** 10.3390/ijerph120606669

**Published:** 2015-06-12

**Authors:** Evelina Pappa, Simela Chatzikonstantinidou, George Chalkiopoulos, Angelos Papadopoulos, Dimitris Niakas

**Affiliations:** 1 Faculty of Social Sciences, Hellenic Open University, Riga Fereou 169 & Tsamadou, Patras 26222, Greece; E-Mails: simelax@gmail.com (S.X.); g.chalkiopoulos@amaliada.gr (G.C.); docpapado@yahoo.gr (A.P.); niakas@eap.gr (D.N.); 2 Department of Internal Medicine, “Attikon” University Hospital, 1 Rimini Street, Athens 12462, Greece

**Keywords:** HRQL, Roma, socio-economic factors, housing conditions, Greece

## Abstract

The aim was to assess the health-related quality of life (HRQL) of the Roma and further to detect the significant determinants that are associated with their HRQL. The cross-sectional study involved 1068 Roma adults living in settlements (mean age 36). HRQL was measured by the Greek version of SF-36 Health Survey and further socio-demographic characteristics (sex, age, marital status, education, permanent occupation *etc*.) and housing conditions (stable housing, access to basic amenities such as drinkable water, drainage, electricity which compose material deprivation) were involved. Non parametric tests and multiple linear regression models were applied to identify the factors that have significant association with HRQL. After controlling for socio-demographic characteristics, health status and housing conditions, sex, age, education, chronic diseases, stable housing and material deprivation were found to be significant determinants of the Roma’s HRQL. Men reported significantly better health than women as well as those who attended school compared to the illiterate. Chronic diseases were remarkably associated with poor HRQL from 10 units in MH (Mental Health) to 34 units in RP (Role Physical). Material deprivation was related to lower GH (General Health), and VT (Vitality) scores and higher RP (Role Physical) and RE (Role Emotional) scores. Chronic conditions and illiteracy are two key areas that contribute significantly to worse HRQL. Policies should be part of a comprehensive and holistic strategy for the Roma through intervention to education, housing and public health.

## 1. Introduction

The Roma population is the largest ethnic group in Europe and it represents one of the most vulnerable and most marginalized groups, facing severe discrimination and social exclusion. As Foldes and Covaci pointed out [1], notable developments in research on the Roma health have been made during recent years by extending the focus from communicable to non-communicable and chronic diseases and also by moving to more analytical studies exploring the factors that determine the health inequalities the Roma face. Available data have shown that the Roma report poor health compared to non-Roma, exhibit less favorable health habits and high risk of diseases, experience lower levels of education, unemployment, live in deprived areas under inhuman living conditions [2,3,4,5,6,7] or experience barriers in accessing health services [8]. It has further been identified that their low socio-economic status fully explains the poor health status but partially the unhealthy behaviors [9].

The European Union has been more considerate for the Roma during the last decade, highlighting the Roma issues in the human rights, discrimination and social desegregation agenda. A Roma framework for national integration strategies up to 2020 has been formed [10] asking the EU Member States to be committed to detecting the multiple problems and promoting the social inclusion of the Roma population. 

In Greece, the Roma population is estimated to be approximately 175,000 [11]. According to the National Strategic Framework for the Roma [12], the Roma population is subject to multiple forms of social exclusion—in the areas of housing, employment, health and education. The four regions where Roma live in higher concentrations are Eastern Macedonia-Thrace, Thessaly, Western Greece and Central Macedonia. Generally, the vast majority of the Roma are marginalized, living on the outskirts of the inhabited areas apart from a small portion which is integrated into the Greek society. Available studies on health have shown that they experience higher prevalence of preventable communicable diseases such as HAV/HBV, greater levels of psychiatric symptoms [13,14] or higher limitations in their daily lives [15]. However, the impact of socio-economic characteristics that may impair their health has not been studied. 

Based on the literature, the aim of the present study is to identify to what extent socio-economic and living conditions explain the Roma’s poor self-assessed health. In this framework, we assessed their health-related quality of life and further explored the relationship between socio-economic factors, housing conditions and their health status. A short testing for validity and reliability of the SF-36 in the Roma population was also applied. 

## 2. Methods

### 2.1. Study Design

A cross-sectional study between December 2010 and April 2013 took place in four municipalities from each region, Thessaly, West Greece, Central Macedonia and East Macedonia-Thrace and involved 1068 Roma adults living in settlements. Inclusion criteria were age >18 years old and adequate level of understanding the Greek oral speech. The study sample consisted of Roma individuals who visited the Social Medical Centers, recently transformed to Centers for Roma and Vulnerable groups (centers operated at local level— in many cases inside the settlements—aiming to detect their health and social problems, inform on primary health issues and access to health services, run programs for social integration and help them with legal issues and contacts with public authorities. The centers are staffed with professionals such as doctors, health visitors, psychologist, social workers and Roma mediators). 

Eligible individuals were randomly selected and representative samples of the Roma living in settlements in each municipality were taken. Face to face interview was conducted in Greek language by the social workers who worked in Social Medical Centers. All participants were informed for the purpose of the study and only 45 Roma individuals refused to participate (response rate 95.5%). Participants were asked to respond to the questionnaire consisting of various questions about their health-related quality of life, socio-demographic characteristics, residing and living conditions, utilization of health services. The study was approved by the Ethical Committee of the Hellenic Open University.

### 2.2. Measurements

HRQL was measured by the SF-36 Health Survey which is a generic, self-administered, multi-item questionnaire measuring HRQL. It is widely used in health services research to record functional health status and general health perceptions. It consists of eight scales: Physical Functioning (PF), Role limitations due to Physical problems (RP), Bodily Pain (BP), General Health (GH), Vitality (VT), Social Functioning (SF), Role limitations due to Emotional problems (RE) and Mental Health (MH). It is measured in a 0–100 range scale with higher scores reflecting better-perceived health [16]. The SF-36 was validated in previous studies in Greece [17,18]. 

The existence of chronic diseases (yes: 1/no: 0) further measured their health status. Demographic and socio-economic characteristics such as sex (male: 1/female: 0), age, marital status (partner: 1/no partner: 0), education (illiterate, primary, secondary education), monthly family income divided into income categories, number of children, the number of roommates, the existence of stable house the last 3 years (yes: 1/no: 0) and housing conditions regarding their access to electricity, drinkable water and indoor bath/drainage were also investigated. The housing conditions were merged into an index which is ranged from 0 (total access) to 3 (non-access to all) and form the variable of the material deprivation.

### 2.3. Statistical Analyses 

First, frequencies were provided in order to describe the sample distribution. Then we applied scale internal consistency assessed by Cronbach’s alpha, item convergent validity and item discriminant validity in order to test the reliability and validity of the SF-36 questionnaire in Roma population. Next we used non parametric tests of independent samples Mann-Whitney and Kruskal Wallis so as to detect significant differences in eight scales according to the various socio-demographic characteristics. Finally, we applied multiple linear regression models, tested for potential collinearity problems, in order to identify the key variables that were significantly related to their HRQL. A two-tailed *p* value of less than 0.05 was considered to be significant. All the statistical analysis was undertaken with the SPSS v17 software.

## 3. Results

An extended description of the socio-demographic characteristics of the sample is provided in Table 1. The Roma is a young population with mean age of 36 years old and the majority (82%) is married, having mainly more than three children. The majority (90.6%) reported stable housing for the last three years, 71.2% reporting above five roommates, while a remarkable rate of 12.5% reported above 1000 euros. A significant rate up to 39% reported no access to one of the three amenities such as drinkable water, electricity and indoor bath/drainage.

**Table 1:** Descriptive characteristics of the study population.

Variables	N	%
**Sex**		
Men	519	48.6
Women	549	51.4
**Age**		
18–24	242	22.7
25–34	347	32.5
35–44	201	18.8
45–54	146	13.7
55–64	97	9.1
65+	35	3.3
**Marital status**		
Single	100	9.4
Married	876	82.0
Divorced/Window	92	8.6
Number of Children		
None	91	8.5
1	87	8.1
2	125	11.7
3	194	18.2
4	264	24.7
5+	307	28.7
**Education**		
No school	631	59.1
Primary	362	33.9
Secondary	75	7.0
**Monthly income (Euro)**		
0–200	190	18.0
201–500	296	28.0
501–800	288	27.3
801–1000	150	14.2
1001+	132	12.5
**Stable housing**		
Yes	968	90.6
No	100	9.4
Number of roommates		
1	21	2.0
2	67	6.3
3	82	7.7
4	138	12.9
5+	760	71.2
**Electricity**		
Yes	832	78.0
No	235	22.0
**Water**		
Yes	895	80.4
No	209	19.6
**Bathroom/Drainage**		
Yes	651	61.1
No	415	38.9
**Chronic diseases**		
Yes	580	54.3
No	387	36.2
Missing	101	9.5

Results of the tests of item internal consistency and item discriminant validity as well as Chronbach alpha for measuring validity and reliability of the questionnaire in Roma population respectively are provided in Table 2. Significantly higher item-scale correlations between items and their hypothesized scales than with competing scales were observed. The 0.40 item-scale correlation criterion was satisfied, confirming 100% item convergence in 34/34 tests for SF-36 scales. Accordingly, item discrimination was successful (when correlation between an item and its own scale is significantly higher- ˃2 standard errors- than with other scales) in 245/245, with scaling success rate in 100%. Finally, internal consistency, measured by Cronbach’a, ranged from 0.78 (GH) to 0.93 (RP) exceeding the 0.70 standard for group level comparisons in all scales [19].

**Table 2:** Summary results of scaling assumptions tests and Conbach a test.

	Item-Internal Consistency			Item-Discriminant Validity		Reliability
	N *	Range of Correlations ^†^	Success/Total ^‡^	Range of Correlations ^§^	Success/Total	Cronbach’ a
PF	10	0.51–0.85	10/10	0.22–0.60	70/70	0.935
RP	4	0.76–0.92	4/4	0.38–0.65	28/28	0.914
BP	2	0.87–0.87	2/2	0.42–0.64	14/14	0.926
GH	5	0.38–0.69	4/5	0.20–0.65	35/35	0.780
VT	4	0.57–0.63	4/4	0.33–0.60	28/28	0.803
SF	2	0.65–0.65	2/2	0.46–0.62	14/14	0.785
RE	3	0.74–0.82	3/3	0.36–0.55	21/21	0.885
MH	5	0.45–0.70	4/4	0.17–0.64	35/35	0.804

* Number of items and number of internal consistency tests per scale; **^†^** Range of correlations between item and hypothesized scale corrected for overlaps; **^‡^** Number of correlations exceeding the 0.40 standard/total number of correlations; **^§^** Range of correlations between items and other scales; Number of successful discriminant validity tests/total number of discriminant validity tests; PF = Physical Functioning, RP = Role Physical, BP = Bodily Pain, GH = General Health, VT = Vitality, SF = Social Functioning, RE = Role Emotional, MH = Mental Health.

### Health-Related Quality of Life

Table 3 presents the distribution of HRQL according to socio-demographic and housing conditions. Significant differences were found among the Roma with men, younger and those having partner reporting higher scores compared to women, older and those without partner in all scales. 

**Table 3:** SF-36 eight scales according to socio-demographic characteristics and housing conditions.

Variables	PF	RP	BP	GH	VT	SF	RE	MH
**Total**	67.61	52.99	57.45	45.65	45.02	55.59	52.99	47.09
**Sex**								
Men	74.41	59.15	63.39	48.90	49.83	59.89	61.04	49.94
Women	61.18	47.17	51.84	42.57	40.48	51.52	45.41	44.40
sig.	***	***	***	***	***	***	***	***
**Age**								
18–24	79.83	68.69	69.63	54.33	53.45	64.82	64.82	63.77
25–34	73.34	60.01	60.20	47.46	46.22	58.28	58.28	55.23
35–44	68.45	51.61	56.13	43.77	44.52	56.71	56.71	54.06
45–54	57.77	38.01	47.24	39.36	39.41	47.26	47.26	44.74
55+	39.73	24.43	41.18	34.77	33.40	39.10	39.10	34.84
**Marital status**								
No partner	57.20	39.11	51.50	41.49	41.13	48.05	39.60	43.62
Partner	69.85	56.11	58.92	46.55	45.92	57.32	56.20	47.93
sig.	***	***	***	***	***	***	***	***
**Education**								
None	58.16	43.97	51.47	39.92	38.59	50.17	49.65	43.30
Primary	80.11	64.01	64.54	51.49	51.98	61.74	56.81	50.38
Secondary	86.80	75.66	73.57	65.66	65.53	71.50	62.66	63.14
sig.	***	***	***	***	***	***	***	***
**Stable housing (last 3 years)**								
Yes	68.08	53.69	58.08	46.09	45.20	56.35	54.13	47.56
No	63.00	64.25	51.40	41.36	43.30	48.25	42.00	42.56
sig.	ns	ns	ns	ns	Ns	*	*	*
**Electricity**								
Yes	69.21	53.81	59.67	48.75	47.69	56.68	61.80	48.38
No	61.80	49.89	49.42	34.48	35.38	51.54	57.02	42.36
sig.	***	ns	***	***	***	***	ns	***
**Water**								
Yes	68.99	54.10	59.37	48.38	47.57	56.59	52.11	48.10
No	61.93	48.44	49.59	34.43	34.56	51.49	56.61	42.94
sig.	*	ns	***	***	***	*	ns	***
**Bathroom/Drainage**								
Yes	68.65	51.11	54.54	48.19	47.96	54.72	48.07	49.94
No	65.98	55.78	62.09	41.59	40.42	56.95	60.56	42.52
sig.	ns	ns	***	***	***	***	***	***
**Monthly income (Euro)**								
0–500	66.31	50.21	59.35	47.06	46.99	54.00	49.86	48.38
501–1000	68.29	55.65	56.08	44.99	43.05	56.19	55.02	44.76
1001+	70.72	54.39	54.76	42.22	45.15	58.39	55.98	49.16
sig.	ns	ns	ns	ns	*	ns	ns	*

sig:* *p* < 0.05; ** *p* < 0.01; *** *p* < 0.001, ns = non significant; PF = Physical Functioning, RP = Role Physical, BP = Bodily Pain, GH = General Health, VT = Vitality, SF = Social Functioning, RE=Role Emotional, MH = Mental Health.

The illiterate have poorer health than those who have attended any level of education whereas higher family income is related to higher scores, but the results are statistically significant only for VT and MH scales. Having stable housing for the last 3 years increases the self-assessed health. Similarly, access to basic material resources, *i.e.*, electricity, indoor bath/drainage and drinkable water are related to higher scores in the majority of the scales. 

Multiple linear regression models (Table 4) were applied to explore significant relationships with the eight SF-36 dimensions. Demographic characteristics were profoundly associated with the Roma HRQL. Males reported significantly higher scores than females, while an inverse relationship between age and HRQL existed, with the older Roma reporting poorer self-assessed health. Marital status had moderate relationship with four out of eight scales. After controlling for demographic variables, significant differences were found according to the socio-economic characteristics such as education. Higher educational level was found to be related to higher HRQL ranging from 3.94 in SF to 8.43 in PF. On the other hand, family income was inversely associated with three scales BP, GH and VT, where higher family income was related to lower HRQL. 

**Table 4:** Multiple linear regression coefficients according to socio-demographic characteristics and living conditions.

Variables	PF	RP	BP	GH	VT	SF	RE	MH
**Gender (Male/female)**								
Non standardized B Coefficients	8.882 ***	8.341 **	7.594 ***	3.278 *	6.826 ***	5.564 **	11.152 ***	3.915 **
Standardized Beta coefficients	0.152	0.094	0.114	0.067	0.150	0.093	0.197	0.092
**Age (10-years group)**								
Non standardized B Coefficients	–5.385 ***	–5.434 **	–3.475 ***	–1.180 *	–1.596 **	–2.531 ***	–3.918 ***	–0.101
Standardized Beta coefficients	–0.248	–0.165	–0.140	–0.065	–0.094	–0.115	–0.118	0.007
**Marital status (partner/no partner)**								
Non standardized B Coefficients	7.835 ***	10.419 **	4.997	3.692 *	3.081	4.143	8.753 *	2.824
Standardized Beta coefficients	0.101	0.088	0.056	0.057	0.051	0.052	0.073	0.050
**Education**								
Non standardized B Coefficients	8.424 ***	6.670 **	4.680 **	4.379 ***	6.926 ***	3.949 **	3.650	5.111 ***
Standardized Beta coefficients	0.180	0.094	0.087	0.112	0.189	0.083	0.051	0.150
**Income**								
Non standardized B Coefficients	–0.861	–0.933	–4.916 **	–3.560 ***	–3.086 **	0.220	0.368	–1.610
Standardized Beta coefficients	–0.021	–0.015	–0.102	–0.102	–0.094	0.005	0.006	–0.053
**Stable housing (yes/no)**								
Non standardized B Coefficients	6.675 *	8.345	9.632 **	4.746 *	2.563	6.916 *	11.482 *	4.554 *
Standardized Beta coefficients	0.068	0.056	0.086	0.058	0.033	0.069	0.076	0.064
**Material deprivation**								
Non standardized B Coefficients	0.264	1.837 *	0.391	–2.091 ***	–1.828 ***	0.473	3.637 ***	–0.721
Standardized Beta coefficients	0.013	0.060	0.017	–0.125	–0.177	0.032	0.118	–0.050
**Chronic Diseases (yes/no)**								
Non standardized B Coefficients	–23.071 ***	–34.565 ***	–24.052 ***	–23.948 ***	–15.285 ***	–22.489 ***	–24.292 ***	–10.202 ***
Standardized Beta coefficients	–0.388	–0.382	–0.353	–0.482	–0.328	–0.370	–0.265	–0.236
**R^2^**	**0.423**	**0.279**	**0.239**	**0.364**	**0.288**	**0.232**	**0.170**	**0.127**

* *p* < 0.05; ** *p* < 0.01; *** *p* < 0.001; PF = Physical Functioning, RP = Role Physical, BP = Bodily Pain, GH = General Health, VT = Vitality, SF = Social Functioning, RE = Role Emotional, MH = Mental Health; Education: illiterate, primary, secondary education; Income: very low/low (up to 500 euros), low/medium (501–1000 euros), high (1001+ euros); Material deprivation: ranged from 0 (total access) to 3 (non-access to all). Values of categorical variables: 1 = male, 0 = female; 1 = partner, 0 = no partner; 1 = yes, 0 = no.

Having stable housing or being under material deprivation were two factors significantly associated with HRQL. Actually, higher material deprivation is related to lower GH, and VT scores and higher RP and RE scores. Finally, the existence of chronic diseases was substantially related to lower HRQL varying approximately from 10 units in MH to 34 units in RP.

## 4. Discussion

The assessment of HRQL of the Roma and the detection of the relationship between socio-demographic characteristics, housing conditions and health status were the purposes of this study. It was involved also the testing of validity and reliability of the SF-36 questionnaire for use in the Roma health surveys. The results identified the validity and reliability of the SF-36 questionnaire in Roma population, whereas various socio-demographic characteristics, material deprivation and chronic diseases were significantly associated with HRQL. 

The SF-36 questionnaire was proved to be reliable and valid for the Roma population as well and therefore could contribute to cross-cultural comparisons. Our findings revealed excellent convergent and discriminant validity. Reliability was also confirmed via the internal consistency and Cronbach’s a exceeded the 0.70 criterion in all scales for the group-level comparisons. The results are comparable with those of previous study in Greek non-Roma general population [17]. 

Sex inequalities occur and women reported worse HRQL compared to men, a pattern that is common either in mixed Roma non-Roma health surveys [20] or in general health population surveys in Greece [21]. It is worth mentioning that in a highly disadvantaged and vulnerable population group such as the Roma, women bear a bigger load which cannot be attributed only to sex. On the contrary, the structure of the Roma community, the role of women, cultural aspects and beliefs in connection with socio-economic factors may explain health disparities between sexes. Furthermore younger age and the existence of partner improve significantly HRQL. 

Education was substantially related to HRQL after adjusting for socio-economic characteristics and housing conditions. Education is a strong social determinant of the Roma HRQL whereas income seems to have a bipolar relationship with the eight dimensions, a finding which needs further research. The association between education and self-assessed health in the Roma has been well established elsewhere [9]. Our results have shown that a rise in educational level improve their self-assessed health from approximately 4 to 8 units and more. In addition to this, the prevalence of chronic diseases is higher in low educational level (data not shown). 

The role of education in health research is well established as a significant determinant for bad health. Education is the pre-condition for future well-being, good jobs and income. In the Roma case, the role of education is crucial. Sixty percent of the respondents in the present study are illiterate. School attendance, completion of compulsory education and school dropout are important issues for the young Roma adults. According to the FRA survey [15], in Greece, more than 35% of the Roma children aged 7–15 haven’t attended school or approximately 9 out of 10 Roma surveyed live in households at risk of poverty. The poverty the majority of the Roma families face is the obstacle for their children to continue school in order to supplement the family income and therefore to break the vicious circle of poverty-illiteracy-bad health.

Stable housing and material deprivation were proved to be considerable factors of the Roma HRQL. Our result shown than having stable housing (for the last 3 years), contributes to substantial improvement in HRQL from 4.5 to above 11 units. It is known that the stability of residence is related to a large degree with the availability of employment. Many Roma families are constantly moving from one region to another in order for seasonal work reasons. On the other hand, as the WHO [22] has pointed out, inadequate housing conditions are associated with risk factors and health hazards affecting the disadvantage groups disproportionally. Lack of access to basic amenities for their living (drinkable water, indoor bath, electricity), which in our study is ranged from 20% to 38%, affects HRQL diversely decreasing GH and VT, but increasing RP and RE, a finding which is difficult to be explained and more evidence is needed. 

Finally, chronic diseases are remarkably associated with poor HRQL. Previous studies have related the chronic diseases with the socio-economic background and have shown that chronic diseases as well as infectious diseases are associated with the social exclusion of the Roma, the unfavorable housing conditions and the inadequate nutrition [14,23]. On the other hand, chronic diseases are related to unhealthy behaviors such as smoking, alcohol consumption *etc*. which are partially explained by the low socio-economic status while culture must also be taken into consideration [9]. As it has been summarized in a recent study [24] the Roma have considerably higher prevalence of unhealthy habits which are connected to poorer health. 

The common model of investigating the Roma’s health and health differences is health studies between the Roma and the non-Roma in order to explore whether the social disadvantage affects the Roma compared to the non-Roma population and further whether ethnicity is confounded with SES [9]. In our study, it was found that the social gradient is significant within the Roma community and that affects their physical and mental health state considerably. In our case, the element of ethnicity does not exist since the target group was Roma only, but the social disadvantages do exist. 

### 4.1. Strengths and Limitations

This study adds to the existing knowledge highlighting the role of certain factors in exploring the explanatory mechanism of the detected associations. Furthermore, the use of SF-36 questionnaire in ethnic groups such as the Roma contributes to implementation of cross-cultural comparisons and to collection of ethnically disaggregated data in health surveys.

Limitations of our study concern first, the cross-sectional nature of our study. Additionally, the sample was drawn from four prefectures of Greece which have the biggest Roma population density, but it is not nationally representative of the Roma. Furthermore, the majority of the studied population lives in settlements having stable housing for the last three years. Nevertheless, travelers and those who live in camps were under-represented. Finally, another limitation lies in the methodology. Monthly family income was not adjusted to the number of persons in the household, due to the ordinal type of the relative question, in order to provide the same living standards. Consequently, this may explain the results we found concerning the association between HRQL and income. 

### 4.2. Recommendations

The lack of accontrol group from the general population did not permit us to further extend our study to the role of ethnicity. Comparative health surveys in both Roma and non-Roma populations are needed in order to assess the impact of social gradient on HRQL in each group. Moreover questions relating to health habits as well as their cultural beliefs should be included in future studies so as to have a wider perception of the Roma’s health profile and to assess the potential impact these factors could have on HRQL.

## 5. Conclusions

The Roma is the most vulnerable ethnic group facing multiple deprivation which encompasses serious inequity. Non-access to basic amenities along with lack of access to education and considerable prevalence of chronic diseases are the background causes which are considered unfair and unacceptable. Sex inequalities are also obvious. Policies should be part of a comprehensive and holistic strategy for the Roma through intervention to education, housing and public health. As it has been noticed, strong political commitment, inter-sectorial coordination and adequate financing are required [25] in order to eliminate the social and health disparities in Roma population. 
